# Recent advances and research progress on microsystems and bioeffects of terahertz neuromodulation

**DOI:** 10.1038/s41378-023-00612-1

**Published:** 2023-11-14

**Authors:** Meiting Liu, Juntao Liu, Wei Liang, Botao Lu, Penghui Fan, Yilin Song, Mixia Wang, Yirong Wu, Xinxia Cai

**Affiliations:** 1 State Key Laboratory of Transducer Technology, Aerospace Information Research Institute, Chinese Academy of Sciences, Beijing, 100190 China; 2 School of Electronic, Electrical and Communication Engineering, University of Chinese Academy of Sciences, Beijing, 100049 China

**Keywords:** Electrical and electronic engineering, Biosensors

## Abstract

Terahertz waves can interact with the nervous system of organisms under certain conditions. Compared to common optical modulation methods, terahertz waves have the advantages of low photon energy and low risk; therefore, the use of terahertz waves to regulate the nervous system is a promising new method of neuromodulation. However, most of the research has focused on the use of terahertz technology for biodetection, while relatively little research has been carried out on the biological effects of terahertz radiation on the nervous system, and there are almost no review papers on this topic. In the present article, we begin by reviewing principles and objects of research regarding the biological effects of terahertz radiation and summarizing the current state of related research from a variety of aspects, including the bioeffects of terahertz radiation on neurons in vivo and in vitro, novel regulation and detection methods with terahertz radiation devices and neural microelectrode arrays, and theoretical simulations of neural information encoding and decoding. In addition, we discuss the main problems and their possible causes and give some recommendations on possible future breakthroughs. This paper will provide insight and assistance to researchers in the fields of neuroscience, terahertz technology and biomedicine.

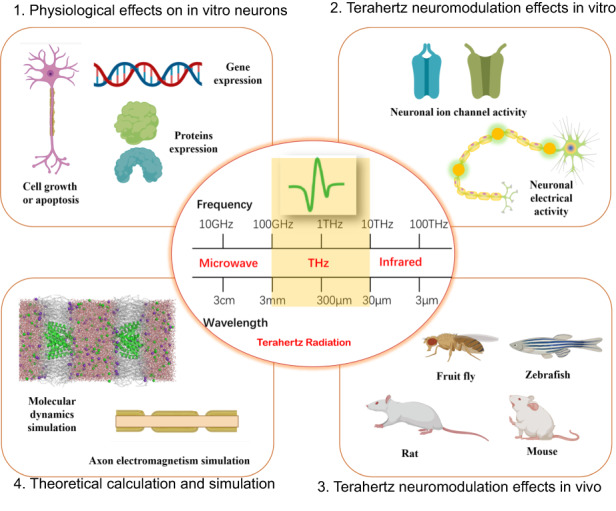

## Introduction

### Importance of research on microsystems and bioeffects of terahertz neuromodulation

Terahertz (THz) science and technology have been challenging but exciting topics in the areas of information science and electronics technology in recent years. The terahertz frequency region occupies a large part of the electromagnetic (EM) spectrum located between the infrared (IR) and microwave (MW) regions^1^. Since terahertz technology was proposed in the 1970s, it has long been called the “terahertz gap” due to the lack of corresponding emission and detection technology^2^. In the 1980s, with the development of ultrafast laser technology as well as the science and technology of semiconductors, an effective method was provided for the generation and detection of terahertz electromagnetic waves^3,4^. Terahertz technology has made unprecedented progress, especially in application fields such as security testing, biomedicine, and communications^5–7^.

Research on the effects of terahertz neuromodulation on the nervous system is a typical representative of the interdisciplinary frontier of information and life sciences, with great strategic significance and broad application prospects. The use of terahertz waves in the medical field mainly stems from the following highly advantageous characteristics^6–8^: Terahertz waves can penetrate materials such as plastics and ceramics and can also detect information on biological tissue under the epidermis. Terahertz waves, with low photon energy, do not have the ionizing effect of X-rays and cause no damage to biological tissues, which have high sensitivity to polar substances. Terahertz wave signals not only have better temporal resolution than microwaves and millimeter waves but also have better spatial resolution. The vibration and rotation energy levels of many biological macromolecules are in the terahertz band; therefore, their conformation, configuration, and response to the environment can be determined by studying their terahertz absorption spectrum.

To date, biomedical research on terahertz waves has mainly focused on two aspects. On the one hand, the characteristics of terahertz spectral information have been studied to obtain characteristic physical parameters (spectral fingerprints), which can then serve as spectroscopic detection markers for biological macromolecules, cells, and pathological or physiological tissues. The terahertz spectrum can be analyzed to identify substances in a sample or to interpret the reaction process, and the spectrum difference or intensity difference combined with certain mathematical methods can also be used to achieve quantitative detection^5,6^. On the other hand, the changes in biological samples after they are irradiated with terahertz waves have been studied to obtain indicators of changes in biological properties and analyze the biological effects and safety of terahertz radiation. When a terahertz wave is directly radiated on biological tissues, it does not cause tissue ionization because its energy level is very low, but it still produces strong interactions with biological tissues. By using this feature, one can understand the operation of biological networks in organisms and then use these biological effects to beneficially regulate vital activities^8–10^. Furthermore, in clinical medicine, terahertz technology can provide a new theoretical basis for the diagnosis and treatment of diseases^11,12^.

### Principles of terahertz radiation effects

Research on the bioeffects of terahertz radiation is an interdisciplinary subject involving information science, electronics and life science. The key technical problem is that the mechanism through which terahertz waves influence living organisms has not been conclusively characterized. Currently, researchers believe that its influence depends on both thermal and nonthermal effects. The thermal effect refers to the effect that radiation exerts on a biological tissue or system by heating it, while the nonthermal effect refers to the effect on a tissue or system that is not directly related to heating after radiation absorption. These effects will be addressed separately.

The propagation of terahertz radiation in living organisms obeys the law of propagation of electromagnetic waves. Water is an indispensable part of biological activity and is also the main chromophore in the terahertz band. The stretching and bending vibrational modes of hydrogen bonds between water molecules are in the terahertz frequency domain. These vibrational modes of water promote strong absorption of terahertz waves. Due to its high moisture content, biological tissue has a strong ability to absorb terahertz radiation and will convert the absorbed radiant energy into heat energy. If there is no photochemistry or phase change process, this will directly cause an increase in the temperature of the object. Therefore, high-power terahertz radiation is more likely to cause thermal effects in biological materials^11,13^.

The nonthermal effects of terahertz radiation on biological systems have always been a controversial topic. The principle of nonthermal effects was proposed by scientists as early as the 1970s^14^. They believe that there are both resonance and nonresonance excitations in the interaction between terahertz waves and biological systems, and resonance excitations involve the energy of terahertz photons. Responses to low-frequency excitations include molecular rotation, lattice vibration, and the acceleration of free carriers^15^. On the other hand, the excitation energy is much greater than the energy of terahertz photons^16^. The nonthermal effect of terahertz radiation is mainly caused by the vigorous oscillation and ultimate breakage of bonds in biological molecules. Experiments have shown that terahertz radiation mainly interacts with the hydrogen bonds in biomolecules, causing low-frequency intramolecular vibrations and resulting in protein conformation changes^17,18^. There are also theoretical models that show that terahertz radiation can directly interact with biomolecules through a nonlinear resonance mechanism to induce coherent excitation and thus produce nonthermal effects^19^.

### Objects of research on the bioeffects of terahertz radiation

The interdisciplinary frontier of information science and life health has consistently been one of the most significant research fields in the twenty-first century. The emergence and application of terahertz technology has greatly promoted the progress of this research. In research on the bioeffects of terahertz radiation, two main factors are considered, namely, the parameters if terahertz radiation (frequency, power, radiation time, etc.) and the composition and characteristics of biological samples.

At this stage, the main objects of research on the bioeffects of terahertz waves include organisms, isolated tissues, cells and biological macromolecules. At the organism level, terahertz radiation can speed up burn repair, increase the level of fibrinolytic factors, and reduce platelet aggregation^20^. At the cellular level, low-intensity terahertz radiation can promote cell proliferation, while high-intensity radiation can cause changes that visibly affect a cell’s interaction with its environment, including changing the stress response mechanism or causing cell death^21,22^. For cell membranes, terahertz radiation increase the surface fluidity and recognition ability and may sometimes damage the membrane^22,23^. At the level of biological macromolecules, terahertz radiation can cause changes in the structure and function of enzymes. Under certain conditions, terahertz radiation can accelerate the temperature-induced unwinding of DNA by breaking hydrogen bonds^24,25^. Some studies have also shown that terahertz radiation will not adversely affect the structure and function of DNA molecules when the intensity is low^26^. However, the effect of terahertz radiation on the nervous system has been less extensively studied.

Understanding the neural basis of cognition, thinking, consciousness and language is the ultimate challenge for humankind to understand nature and itself^27,28^. Neural stimulation and regulation technology are widely regarded as important tools in neuroscience, neuroengineering and clinical applications. In recent decades, a variety of pioneering neuromodulation methods have emerged, including noninvasive methods such as transcranial magnetic stimulation (TMS), transcranial current stimulation (TCS), transcranial ultrasound stimulation (TUS), electrode deep brain stimulation (DBS), and infrared nerve stimulation^29–33^.

**Fig. 1:**
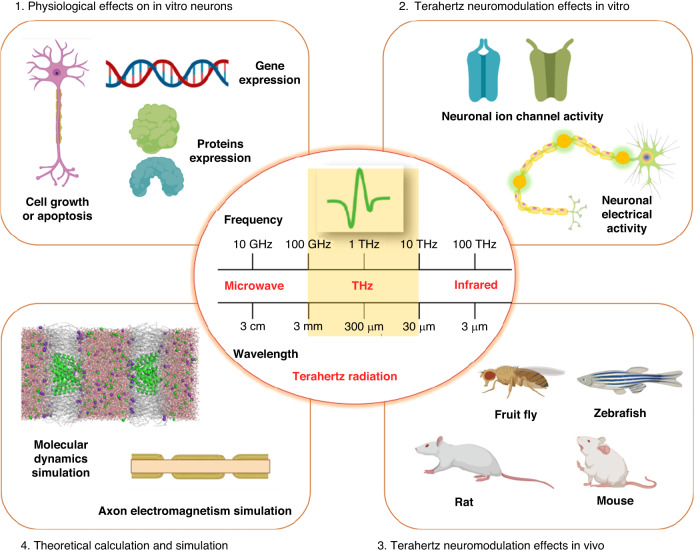
Biological effects of terahertz radiation on the nervous system. Created with http://medpeer.com; some material with permission from^81^. Copyright 2022, American Physical Society

Research regarding the biological effects of terahertz radiation on the nervous system is the interdisciplinary frontier of terahertz technology and brain science; this area of research is also of great interest for information science and has great strategic significance as well as broad application prospects. Terahertz waves can interact with the nervous system of organisms under certain conditions. Compared to common optical modulation methods, terahertz waves have the advantages of reduced photon energy and reduced risks. The use of terahertz waves for brain science will offer a novel technical means for the regulation and detection of the nervous system and become a commanding presence on the frontiers of science and technology; in fact, it has been included in some countries’ national strategic planning. Research in this area has important application prospects not only in the treatment of neurological diseases but also in real-time communication, brain–computer interfaces and new generations of artificial intelligence. In this article, both historical and recent advances in terahertz neuromodulation of the nervous system are reviewed, addressing the bioeffects of terahertz radiation on neurons in vivo and in vitro, novel regulation and detection methods with terahertz radiation devices and neural microelectrode arrays, and theoretical simulations of neural information encoding and decoding. The article also analyzes the current problems and possible future breakthroughs in the field of terahertz neuromodulation, which few previous reviews have addressed. This article will elaborate on four areas, as illustrated in Fig. 1.

## Research on terahertz neuromodulation of the nervous system in vitro

At present, the study of the effects of terahertz radiation on neural tissue is still in its preliminary stage^34,35^. Within this area, studies on isolated cells began earliest and are most abundant. The main objects have been neurons and neuron-like cells of different sources and types. However, different experimental conditions, such as terahertz wave frequency, irradiation time and irradiation power, often produce different results; thus, it is necessary to carry out in-depth comparisons and analysis.

### Bioeffects of terahertz radiation on neurons in vitro

As with all other types of electromagnetic radiation, the safety and potential harmful effects of terahertz waves should be the primary consideration^36^. Many in vitro studies of the physiological effects of terahertz radiation on neurons have been conducted (Table 1). Bourne^37^ conducted a study to evaluate the bioeffects of terahertz radiation (0.14 THz, irradiation time varying from 10 min to 24 h, peak power between 24 and 62 mW/cm^2^) on ND7–23 cells (an immortalized rat DRG/mouse neuroblastoma cell line that can proliferate in the undifferentiated state but can be induced to differentiate and develop neurite-like projections). To evaluate the effects of terahertz radiation on differentiation, glutathione (GSH) and heat shock protein 70 (Hsp70) levels were examined before and after differentiation. The results showed that terahertz radiation did not induce a stress response and resulted in no detectable adverse reactions. Tsurkan^38^ studied the impact of broadband pulsed terahertz radiation (0.05–2.00 THz) on neurite growth in the sensory ganglia of 10- to 12-day chicken embryos. Experiments were conducted with different power densities and exposure times. In the tests with power densities of 5 and 50 μW/cm^2^, no influence on the growth of sensory neurons was observed. However, at a power density of 0.5 μW/cm^2^, the area index of the experimental explants increased by 24% compared to that of the control explants. Therefore, the author inferred that terahertz radiation at a specific power could stimulate cell growth. Xie^39^ studied the apoptotic effects of terahertz radiation on retinal ganglion cells. Mouse retinal ganglion cells were exposed to terahertz radiation (0.1–3.5 THz), and the apoptosis of cells after exposure for 0, 6 and 12 h was detected. The results showed that the apoptosis rates decreased at 6 and 12 h after exposure compared with those of the control group.

**Table 1:** Physiological effects of terahertz radiation on neurons in vitro

Object	Methods	Parameters	Effects	Reference
ND7–23 cells	Examining GSH and Hsp70 levels before and after differentiation	0.14 THz, 24–62 mW/cm^2^, 10 min to 24 h	No stress response and no detectable adverse reactions	^37^
Sensory ganglia of chicken embryos	Evaluating explant neurite growth intensity	0.05–2 THz, exposure time 3 min.	Area index increased by 24% at a power density of 0.5 μW/cm^2^	^38^
Mouse ganglion retinal cells	Detecting the apoptosis of cells after terahertz exposure	0.1–3.5 THz, 5, 10, 20, 30 and 40 min	A certain inhibitory effect on the increase in apoptosis	^39^
NGF treated PC12 cells	Measurement of intracellular and extracellular DA and DOPAC content and DAT expression	0.06 THz, 5 mW/cm^2^, 24 h exposure	No evidence of nonthermal effects observed on dopamine metabolism	^40^
RPE cells, HCE cells and Müller cells	Assessing gene expression profile of cells	0.7 THz, 0.3 mW/cm^2^, 6 h	Obvious cellular gene expression differences between irradiated cells and control cells	^41^
Mouse primary cortical neurons and oligodendrocytes	Patch-clamp recording, TUNEL assay and gene and protein expression assessment	3.1 THz, 70 mW/cm^2^	Increased expression of synaptic proteins and genes	^42^
Rat glial cells	Assessing the changes in mitochondrial membrane potential	0.12–0.18 THz, 3.2 mW/cm^2^	The nonthermal effect of terahertz radiation caused cell apoptosis	^43^

**Fig. 2:**
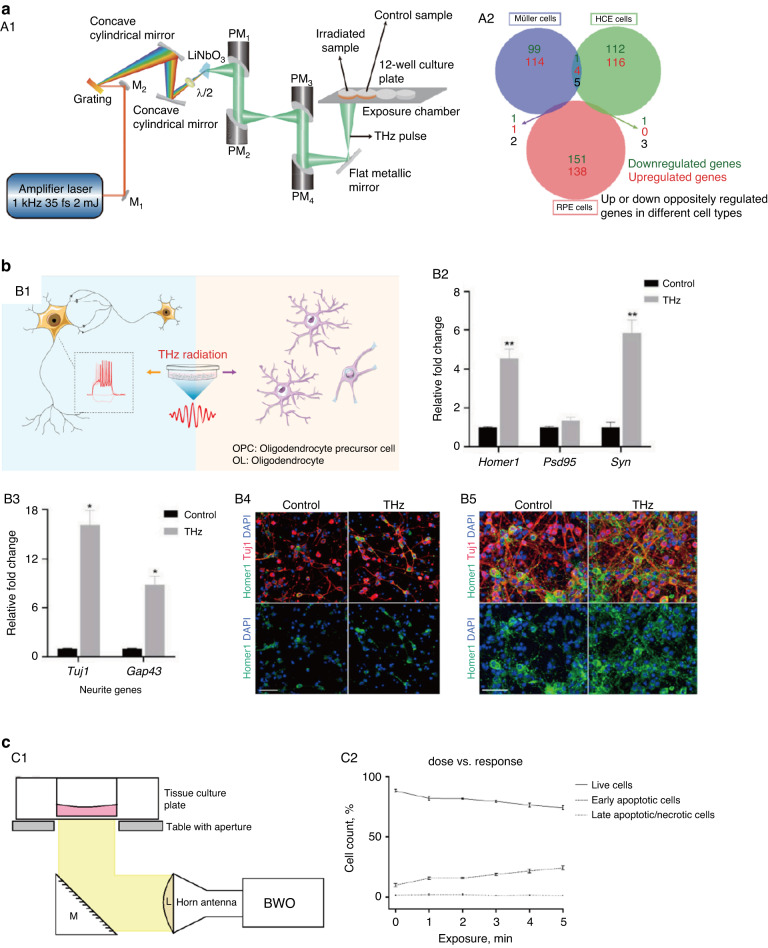
Physiological effects of terahertz radiation on in vitro neurons. **a** Gene expression profile of eye cells under terahertz irradiation. A1. Experimental setup for exposure of cells to terahertz radiation; A2. Venn diagram of gene expression results in these three cell types. Reproduced with permission from ref. ^41^, Copyright 2019, Chinese Physics B. **b** Effects of terahertz radiation on mouse cortical neurons. B1. Schematic of the irradiation setup; B2. Quantitative real-time PCR for three synapse-related genes, Homer1, Psd95, and Synapsin; B3 Quantitative real-time PCR for two neurite growth genes, Tuj1 and Gap43; B4 Representative images of immunostaining of Tuj1 and Homer1 in 6 DIV low-density cultures after THz irradiation; B5 Quantification of the percentage of Homer1^+^ cells within Tuj1^+^ neurons in control and THz-irradiated cultures. Reproduced with permission from ref. ^42^, Copyright 2021, Elsevier. **c** Dose-dependent cytotoxic effect of terahertz radiation. C1. Experimental setup to test the effect of terahertz irradiation on rat glial cells; C2. The number of cells as a function of the THz radiation exposure time. Reproduced with permission from ref. ^43^, Copyright 2017, Optical Publishing Group

Genes are the basic functional unit of inheritance and mutation in organisms, determining or affecting all biological activity, while proteins are the structural basis of cells and tissues and are also important functional components of living systems. Recent studies have shown that terahertz waves can affect the nervous system by regulating gene and protein expression. Haas^40^ investigated the bioeffects of radiation (0.06 THz, with an incident power density of 5 mW/cm^2^, 24-h exposure) on nerve growth factor (NGF)-treated PC12 cells. The results showed that cytosolic dopamine (DA) and 3,4-dihydroxyphenylacetic acid (DOPAC) contents were not significantly modified by exposure to THz for 24 h. However, extracellular accumulation of DOPAC was found to be slightly but not significantly increased. Zhao^41^ studied the gene expression profile of cells exposed to terahertz radiation (0.7 THz) (Fig. 2a). Three different types of eye cell lines were used, including human ARPE-19 retinal pigment epithelial cells (RPE cells), simian virus 40-transformed human corneal epithelial cells (HCE cells), and human MIOM1 Müller cells (Müller cells). Significant differences in cellular gene expression were recorded between all three types of irradiated cells and their control cells. After correction for multiple comparisons using the Benjamin–Hochberg false discovery rate (FDR) method, the expression changes of seven genes were observed after terahertz irradiation, indicating that the effect on gene expression can last for more than 15 h. The results also showed that there was no overlap between differentially expressed genes (DEGs) for the three cell types, suggesting that the bioeffects of terahertz radiation are cell type dependent. Zhao^42^ conducted studies on the effects of terahertz radiation on mouse cortical neurons. A customized quantum cascade laser (QCL) (3.1 THz, 70 mW/mm^2^) was used; the irradiation setup is shown in Fig. 2b. Cultured neurons exhibited significantly increased expression of synapse-associated proteins, e.g., Homerl and Synapsin, with either short- or long-term irradiation. Meanwhile, microarray assays revealed that genes associated with “neuron projection,” “synapse organization,” and “dendritic spine” were upregulated in terahertz-irradiated neurons.

In the above studies, the neurons showed good tolerance, no toxicity and no allergic reaction to terahertz radiation. Histology showed that terahertz radiation did not cause pathological changes. At the same time, low-power irradiation could even promote some physiological functions. However, studies have also shown that terahertz radiation can cause cytotoxic effects in some types of cells. In 2017, Borovkova^43^ demonstrated a dose-dependent cytotoxic effect of terahertz radiation (0.12–0.18 THz, average power density of 3.2 mW/cm^2^). After 1 min of exposure, the number of apoptotic cells increased 1.5-fold, and after 5 min, it reached 2.4 times the initial value (Fig. 2c). They believe the reason is that many intracellular structures of glial cells are obviously polarized, and the size of glial cells is smaller than the wavelength of the terahertz source used. Therefore, to minimize the harmful effects of terahertz radiation, an increasing number of detailed studies are needed to clarify the parameters of terahertz waves and the types of neurons that can safely be subjected to neuromodulation in this way.

### Detection and evaluation of neuronal activity in vitro with a terahertz radiation system

Every cell possesses a resting transmembrane potential. In neurons, the existence of an electrical potential across the cell membrane sustains a chemical gradient between intra- and extracellular spaces, which drives transmembrane transport of organic or inorganic molecules and ions. Various neuromodulatory approaches have been employed to alter neuronal discharge activity and thus regulate brain functions and alleviate neurological disorders (Table 2). Terahertz technology could be a potential approach for neuromodulation because it possesses high temporal and spatial resolution.

**Table 2:** Effect of terahertz radiation on neuronal activity

Object	Methods	Parameters	Effects	Reference
Cultured neurons from 4 rat brain regions and 3 lines of neuron-like cells	Analysis of temperature change, neurite growth, cell membrane roughness, micromorphology, neurotransmitters and synaptic proteins	0.16 THz at 50 mW, 0.17 THz at 10 mW; exposure times of 6 and 60 min	Different cells responded differently to terahertz waves; the effects were closely related to the parameters of terahertz irradiation	^44^
Isolated neurons of *Lymnaea stagnalis*	Basic characterization of cells (morphology, regeneration of neurites, characteristics of electrogenesis)	2.50 THz, 0.3–30 mW/cm^2^, 1–120 min	Blank protrusions of the membrane, disorders of the growth of processes, and a decrease in the membrane potential	^45,46^
Hippocampal neurons from SD rats	Measurement of neuronal membrane potential and intracellular concentrations of ions	0.1 THz; 2.65 mW/cm^2^; exposure times of 5, 15 and 25 min	Promoted neuronal excitation by regulating the concentrations of ions	^47^
Pyramidal neurons in cortical slices from neonatal rats	Assessment of electrophysiological characteristics, cellular membrane permeability and nanoporation	0.06 THz, 40 nW/cm^2^ - 1 mW/cm^2^	Induced changes in neuronal firing and a reduction of membrane input resistance	^48,49^
Primary hippocampal neurons	Bioinformatic analysis	0.141 THz	Enhanced neuronal synaptic plasticity by promoting the growth of neuronal processes and modulating the release of neurotransmitters	^50^
Parapharyngeal ganglia isolated from *Lymnaea stagnalis*	Assessment of cell membrane permeability by fluorescence staining	2.3 THz, 0.5–20 mW/cm^2^, exposure time of 30 s	Damaged membrane integrity but did not lead to cell death	^51,52^
PC12 cells	Assessment of cell membrane permeability	0.3–19.5 THz, 10 min	Promoted nanoparticle uptake by cells without causing apoptosis, necrosis or physiological damage	^53^

Zhao^42^ performed patch-clamp recording to analyze the effects of terahertz radiation on the excitability of oligodendrocyte precursor cells. Two types of neurons with different spike firing modes were observed: delayed firing neurons (DFNs) and regular firing neurons (RFNs). After terahertz exposure, the spike firing of the DFNs increased markedly compared to that of the control group. However, the RFNs did not show a significant difference between the control and terahertz groups. This paper speculated that the enhanced neuronal activity of DFNs might be caused by increased excitatory synaptic transmission but not by intrinsic properties of neurons and that different types of neurons (DFNs and RFNs) might show different degrees of sensitivity to terahertz irradiation. Tan^44^ conducted a study using neurons from four rat brain regions (hippocampus, cerebral cortex, cerebellum, and brainstem) and three neuron-like cell lines (MN9D, PC12, and HT22 cells). Cells were exposed to terahertz radiation using the following parameters: power of 50 mW (0.16 THz) and 10 mW (0.17 THz) with exposure times of 6 and 60 min. The results showed that different cells had different responses to terahertz waves. Primary hippocampal neurons, cortical neurons and MN9D cells are relatively sensitive to terahertz waves, and most cells show inhibitory effects after terahertz waves. At the same time, the regulatory effect of terahertz radiation on neurons is also related to the radiation parameters of terahertz radiation. Olshevskaya^45,46^ studied the effects of terahertz radiation (2.50 THz) on the basic characteristics of the isolated neurons of *Lymnaea stagnalis* (morphology, regeneration of neurites, and characteristics of electrogenesis). It was found that at an average radiation power of 30 mW/cm^2^ and above (1 min of exposure), there was disruption of membrane morphology and intracellular structures as well as the membrane potential of neurons. At lower power levels (1–10 mW/cm^2^), the morphological changes were significantly weakened, and at a power of 0.3 mW/cm^2^, no cellular changes were observed. The results show that the effect of terahertz radiation on cells is directly related to the irradiation power and frequency. These studies demonstrate that the biological effects of terahertz radiation on neurons are closely related to the parameters of terahertz waves and the type of cells; therefore, a great deal of research still needs to be carried out before terahertz radiation is used as a neuromodulatory method.

**Fig. 3:**
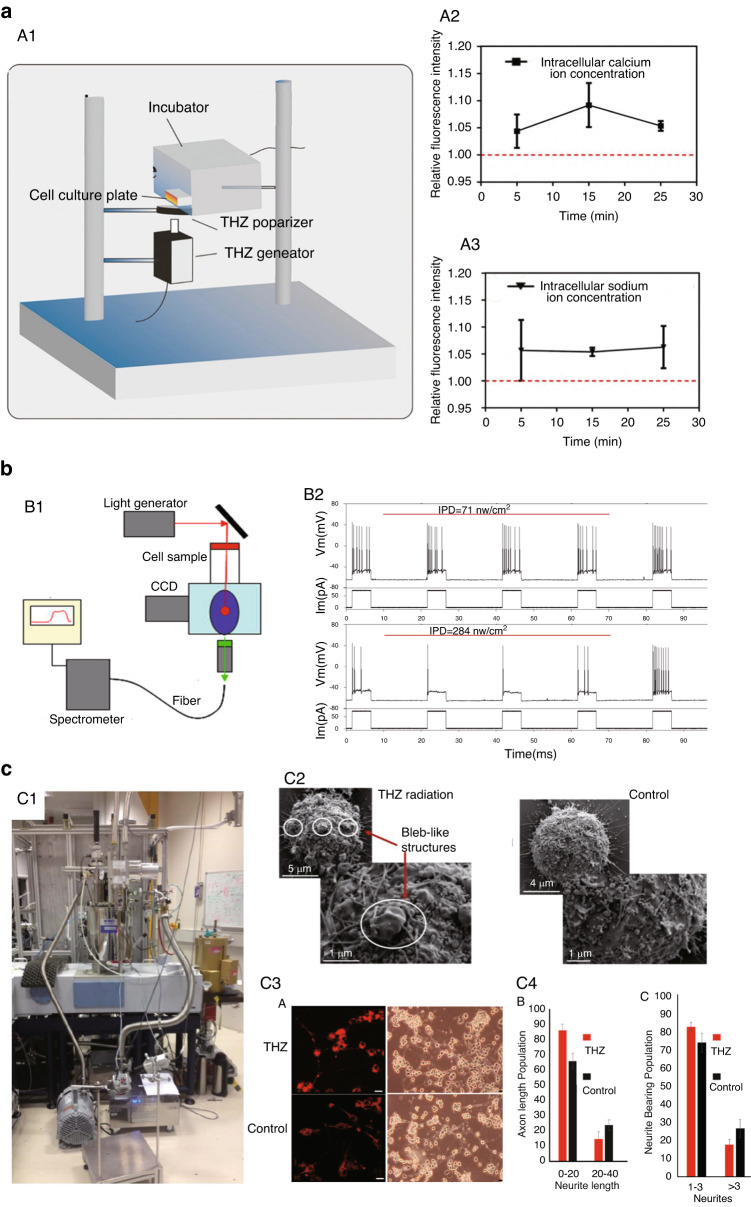
In vitro study of the bioeffects of terahertz radiation on neuronal activity. **a** Effects of terahertz radiation on the excitability of hippocampal neurons in SD rats. A1. Experimental setup to test the effects of terahertz exposure on hippocampal neurons; A2. Fluorescence results reflecting intracellular calcium ions after 5 min, 15 min, and 25 min of irradiation; A3. Fluorescence results reflecting intracellular sodium ions after 5 min, 15 min, and 25 min of irradiation. Reproduced with permission from ref. ^47^, Copyright 2021, Chinese Laser Press. **b** Effect of radiation (0.06 THz, 185 mW) on pyramidal neurons in cortical slices of neonatal rats. B1. Schematic experimental setup used for terahertz irradiation of pyramidal neurons in cortical slices from neonatal rats; B2. Patch-clamp recording results from terahertz-irradiated cells. Reproduced with permission from ref. ^48^, Copyright 2010, SPIE. **c** Effects of terahertz radiation on PC12 pheochromocytoma cells. C1. Experimental setup for terahertz exposure of PC12 cells; C2. Scanning electron micrographs of PC12 cells after 10 min of terahertz radiation exposure; C3. Images of neurite outgrowth after irradiation; C4. Quantification of axon outgrowth and neurite-bearing cell population. Reproduced with permission from ref. ^53^, Copyright 2019, MDPI

Zhang X.^47^ conducted a study to analyze the effects of terahertz radiation (0.1 THz and 2.65 mW/cm^2^) on the excitability of hippocampal neurons in SD rats (Fig. 3a). It was found that terahertz radiation for 15 and 25 min caused significant depolarization of hippocampal neurons, thereby inducing neuronal excitability. Measurement of intracellular ion concentrations showed that the concentrations of Ca^2+^ and Na^+^ in hippocampal neurons increased after terahertz irradiation, while the concentration of K^+^ decreased. Siegel PH^48,49^ examined the effect of radiation (0.06 THz, 185 mW) on pyramidal neurons in cortical slices from neonatal rats (Fig. 3b). Fluorescence microscopy imaging showed that after short-term terahertz exposure, cell membrane permeability and nanopore formation increased. Patch-clamp results indicated that low levels of radiation power (1 μW/cm^2^) could induce changes in neuronal firing and a profound reduction in membrane input resistance, which, in turn, might lead to changes in neuronal excitability. Liu^50^ investigated the biological effects of terahertz waves with a frequency of 0.141 THz on primary hippocampal neurons and the potential mechanisms of these effects. The cells in the sham radiation group were processed in parallel, but the radiation source was turned off. The temperature of the medium was monitored, and no significant changes were observed, indicating that the 0.141 THz waves did not produce a thermal effect. The concentrations of intercellular Ca^2+^ and neurotransmitters in the culture supernatant were analyzed. It was found that terahertz waves increased the intracellular Ca^2+^ concentration and decreased the content of inhibitory neurotransmitters. These studies show that terahertz radiation can control the ion channels of neurons by regulating the concentration of ions in neurons, thereby activating or inhibiting neural activity.

Zapara^51,52^ conducted a study to examine the effect of terahertz radiation on parapharyngeal ganglia isolated from *L. stagnalis*. The results showed that terahertz radiation (frequency of 2.31 THz, average power of 0.5–20 mW/cm^2^ and exposure time of 30 s) did not lead to cell death but caused damage to cell membrane integrity sufficient to allow the penetration of trypan blue into the cell; notably, this damage was reversible. Perera^53^ explored the potential effects of terahertz radiation (frequencies ranging from 0.3 to 19.5 THz for a period of 10 min) on PC12 pheochromocytoma cells in the absence of thermal effects (Fig. 3c). The surface of irradiated PC12 cells formed protruding structures larger than 1 μm in diameter, and the internalization of silica nanospheres (*d* = 23.5 nm) and clusters thereof (*d* = 63.9 nm) increased significantly, which indicated that terahertz radiation could cause increased cell permeability. These findings indicate that terahertz irradiation can promote nanoparticle uptake by cells without causing apoptosis, necrosis or physiological damage; such results provide deeper fundamental insight into the biological effects exerted on cells by environmental exposure to terahertz radiation.

## Research on terahertz neuromodulation of the nervous system in vivo

### Regulation of animal emotion by terahertz radiation

The brain is a complex system. Systematic studies must detect and analyze the brain at multiple scales from neurons to nuclei and from neural circuits to the whole brain. In recent years, scientists have performed a large number of in vivo animal experiments, and the results have shown that terahertz wave stimulation with different intensities, frequencies, and durations can enhance or inhibit neural activity (Table 3).

**Table 3:** Effects of terahertz radiation on living animals

Object	Parameters	Effects	Reference
Albino rats subjected to acute immobilization stress	0.15 THz; 3 mW/cm^2^; 15, 30 or 60 min	Terahertz exposure produced increased levels of depression, and the effects of radiation depend on the duration of exposure.	^54–56^
Mice	3.6 THz, 23.6 mW/cm^2^, 15–30 min	Mice exposed to terahertz radiation for 15–30 min exhibited increased levels of anxiety	^57^
Four groups of mice subjected to a standard formalin test	0.04 to 0.075 THz, power of 2.25 mW, irradiation duration of 10 min	A single session of preventive irradiation of the E-36 AP suppressed somatic pain in mice	^58^
SCA3 transgenic mouse model	43-60 THz, 50 mW/cm^2^, 30 min 3 times per day	Irradiation supported the survival of Purkinje cells in the cerebellum and significantly improved the gait performance of SCA3 transgenic mice	^59^
Mice with AD-like symptoms	30–38 THz, 1 h per day for 6 weeks	Terahertz light administered 1 h per day for 6 weeks could improve learning and memory abilities and decrease Ab plaques in mice with AD-like symptoms	^60^
C57BL/6 mice	0.14 THz for 20 min at a time, once a day for two weeks	Terahertz waves may reduce anxiety- and depression-like behavior and increase social interaction in mice	^61^
SD rats	0.04 THz, 0.01 mW	Exposure suppressed the conduction of pain impulses from the brain meninges to suprasegmental structures of the CNS and induced a short-term decrease in neuronal excitability	^62^
Mouse neocortical slices and larval zebrafish	52.6 THz; 24, 72, 159, 392, 511, 684 and 851 μW/mm^2^	Reversible, long-distance, nonthermal modulatory effects on ion channel activity, neuronal signaling, and sensorimotor behavior	^63^

**Fig. 4:**
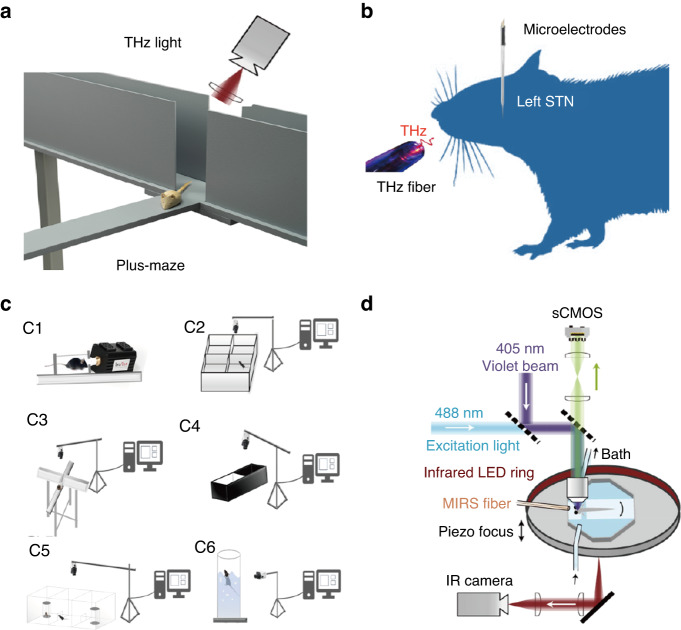
In vivo studies of the bioeffects of terahertz radiation on the nervous system. **a** Schematic drawing of testing the behavioral effects of terahertz radiation using an elevated plus maze designed to test for anxiety (created with http://medpeer.com according to ref. ^54^). **b** Schematic drawing of a study of left STN neuronal activity under THz radiation (created with http://medpeer.com according to ref. ^62^). **c** Schematic drawing of mouse behavioral analysis under terahertz radiation. C1, illustration of terahertz irradiation of C57BL/6 mice; C2–C6, devices for mouse behavior detection. Reproduced with permission from ref. ^61^, Copyright 2022, MDPI. **d** Schematic drawing of the calcium and behavior imaging system under terahertz radiation. Reproduced with permission from ref. ^63^, Copyright 2021, PNAS

Kirichuck^54–56^ was the first to study the effects of terahertz radiation on the nervous system in living animals. The behavioral reactions and biochemical parameters of albino rats subjected to acute immobilization stress under terahertz radiation (3 mW/cm^2^ of 0.15 THz) were studied. It was shown that terahertz waves inhibited upregulation of the sympathoadrenal and pituitary–adrenal axes during acute stress reactions and reduced the levels of catecholamines, corticotropin, and corticosterone in male rats exposed to acute stress. In addition, rats exposed to terahertz radiation for 60 min exhibited increased levels of depression, while rats exposed to durations shorter than 60 min did not exhibit this effect (Fig. 4a). Bondar^57^ conducted a similar study on mice (*n* = 10–12). The results showed that mice exposed to terahertz radiation (3.6 THz, 23.6 mW/cm^2^) for 15–30 min exhibited increased levels of anxiety. These studies used terahertz waves of specific frequencies to irradiate animals in an active state and then indirectly analyze the effects of terahertz waves on the nervous system of living animals through behavioral analysis and detection of biochemical indicators. It was indicated that exposure to terahertz waves affected animal behavior, but the mechanism and physiological responses responsible for these effects are unclear.

### Treatment of animal neurological diseases by a terahertz radiation system

Compared to common optical modulation methods, terahertz waves have the advantages of low photon energy and low risk; therefore, many studies have used terahertz waves as a neuromodulatory tool in the treatment of some neurological diseases. Chuyan^58^ studied the effects of terahertz (4.0–7.5 THz, 2.25 mW, irradiation session of 10 min) wave irradiation of acupoint (AP) E-36 on four groups of mice. It was found that irradiation of AP E-36 provided clear, significant suppression of somatic pain in mice. The author speculated that electromagnetic radiation exerts analgesic effects upon the irradiation of acupoints, which serve as polymodal receptors of environmental sensitivity. Liu^59^ assessed the effect of terahertz (43–60 THz, 50 mW/cm^2^, exposure time of 30 min three times per day) acupoint therapy on spinocerebellar ataxia type 3 (SCA3) in vivo. It was found that radiation significantly improved gait performance in terms of maximal contact area, stride length, and base support in the forepaws, hindpaws or both. Wang^60^ conducted a study to examine whether terahertz radiation (30–38 THz) was effective in mitigating pathology and cognitive function in Alzheimer’s disease (AD). When terahertz radiation was administered 1 h per day for 6 weeks in mice with AD-like symptoms, it improved the learning and memory abilities of the animals and decrease Ab plaques, indicating that different wavelengths of low-energy electromagnetic radiation might have therapeutic potential in AD. Qi^61^ studied behavioral changes in C57BL/6 mice treated with terahertz waves (0.14 THz, 20 min each time, once a day for two weeks) (Fig. 4c). The results showed that terahertz waves could reduce anxiety- and depression-like behavior and increase social interaction in mice. In addition, terahertz treatment did not appear to cause aggression in mice.

### Detecting and evaluating the bioeffects of terahertz radiation in vivo

What happens to the nervous system of living animals under terahertz radiation? Answering this question requires simultaneous detection of neural signals in animals under terahertz radiation. Implantable brain–computer interfaces (IBCIs) can record the discharge signals of multiple neurons in real time, with the advantages of minimal invasiveness and high temporal and spatial resolution. BCIs have developed rapidly and have been widely adopted, providing a new technical means for the study of terahertz neural regulation in living animals.

Kirichuk^62^ recorded the neuronal activity of the left STN in rats with a tungsten microelectrode under terahertz radiation (0.04 THz, 0.01 mW). The results showed that THz exposure suppressed the conduction of pain impulses from the brain meninges to suprasegmental structures of the CNS and induced a short-term decrease in neuronal excitability (Fig. 4b). Liu^63^ conducted systematic research with cutting-edge technologies from different fields, such as patch-clamp recording, molecular simulation, light field imaging, and behavioral analysis (Fig. 4d). With exposure to terahertz radiation (53 THz), zebrafish escape behavior was regulated bidirectionally, becoming weaker under weak sensory stimulation but becoming stronger by strong stimulation. The patch-clamp recording results revealed the cause at the cellular level: the increased potassium current narrows the waveform of the neuron’s output signal—the action potential—and produces a shunting effect, which weakens the neuron’s response to weak stimuli weaker and strengthens its response to strong stimuli.

**Fig. 5:**
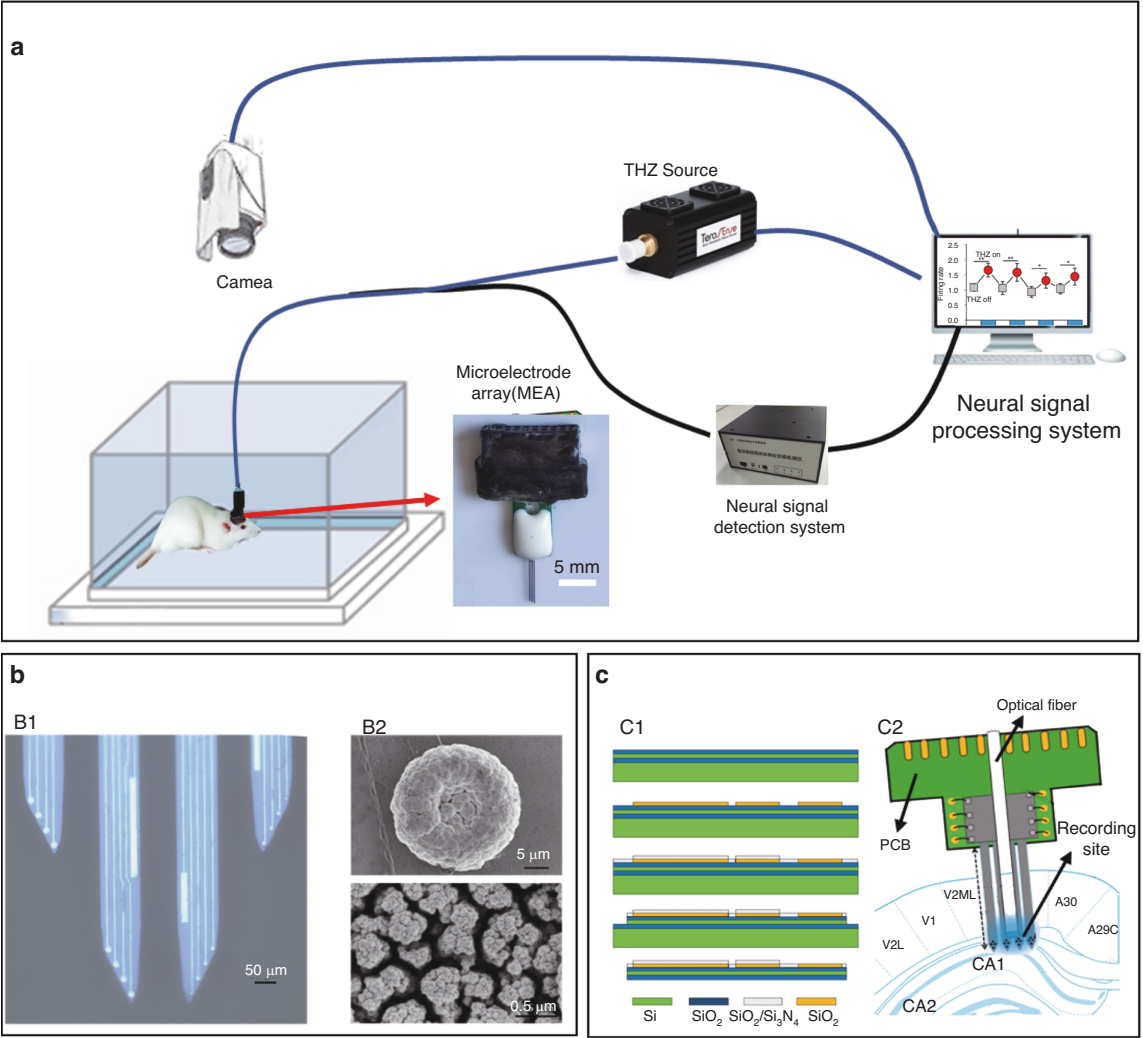
MEA-based study of the effects of terahertz radiation in live animals. **a** Schematic drawing of the MEA-based study of the effects of terahertz radiation in live animals. **b** Microelectrode arrays modified with platinum nanomaterials. B1. Micrograph of the materials of the modified MEAs. B2. Scanning electron microscope (SEM) images of electrode sites. Reproduced with permission from ref. ^66^, Copyright 2020, MDPI. **c** Microelectrode array integrated with an optical fiber. C1. Schematic diagram of the fabrication process; C2. Schematic diagram of the implantation process. Reproduced with permission from ref. ^67^, Copyright 2022, MDPI

Our group fabricated a series of neural microelectrode arrays (MEAs) to record the discharge activity of neurons in vivo^64–67^. We are conducting a study to observe the discharge activity of neurons in the anterior cingulate cortex and the influence of terahertz radiation on brain function with a new MEA incorporating terahertz fibers (Fig. 5). The results show that terahertz radiation significantly increases the spike emission frequency and the local field potential (LFP) power across the whole frequency range. These results indicate that terahertz radiation can stimulate the activity of cortical neurons and produce abnormal excitation in the brain.

To date, terahertz neuromodulation research has been developed from macroscopic analysis of behavior to microscopic neuron detection and from protein and gene analysis to real-time neural activity monitoring. Through the above studies, it has been found that nonthermal, reversible terahertz neuromodulation has potential clinical application value for brain function regulation and brain disease treatment. However, studies on the bioeffects of terahertz waves on large animals are still very scarce and need to be further extended and verified.

## Theoretical calculation and simulation of the bioeffects of terahertz radiation on neurons

### Theoretical calculation of neural information transmission in the terahertz band

Cognitive functions such as reasoning and learning use a number of distinct brain regions in a time-sequenced manner. Neurons conduct electrical impulses from the cell body to other neurons along thread-like axons, and the generation and transmission of nerve signals in organisms produce a physical field. Morgera^68,69^ believes that the function of the brain depends not only on the physical connections between neurons but also on the wireless connections between nervous systems, which are called electric near-field connections.

Liu^70^ conjectured that this physical field should be a high-frequency electromagnetic field from terahertz to infrared. Chang^71,72^ built a quasi-quantum model of amplification for neural terahertz/infrared information at the nodes of Ranvier. Simulations further demonstrated that myelinated nerve fibers serve as dielectric waveguides for high-frequency electromagnetic information in a certain mid-infrared to terahertz spectral range, and the energy for signal propagation is supplied and amplified when the signal crosses the nodes of Ranvier via a periodic relay.

### Ion transmembrane transport simulation of terahertz bioeffects on neurons

Jiang^73^ found that K^+^ channels in biological systems allow extremely fast ion transport (~10^7^ ions/s), ultralow energy consumption and ultralow ionic resistance. He proposed that ion and molecule flows in biological ion channels can regarded as quantum-confined superfluids, which are different from the ion diffusion process in neural networks. In this case, the quantum states of ions and molecules could carry enormous amounts of bioinformation. In the biological K^+^ channel as opposed to the diffusion of K^+^, the terahertz absorption of quantum-confined K^+^ is located at ~1580 and ~1603 cm^−1^ with two sharp peaks^74^. Therefore, neural signals could be studied by the terahertz responses of quantum-confined ions in biological channels.

Song^75^ proposed a cell vibron polariton (cell-VP) in the myelin sheath of nerves, i.e., a collectively coherent mode of a photon and all phospholipid molecules in the myelin sheath. Calculations and analyses indicated that the energy of a terahertz photon at a special frequency (87 THz) can be stored in a cell-VP, where it can be resonantly trapped in the myelin sheath in vivo and promote the conduction of electric impulses in the neuron. Therefore, terahertz radiation might affect the mode strengths and potentially modulate the subsequent neuronal responsiveness, including neural signal generation and conduction.

### Molecular dynamics simulation of the bioeffects of terahertz radiation on neurons

Since molecular dynamics simulation can directly depict the femtosecond change in the cell membrane system at the atomic scale, it has been demonstrated to be an effective method for studying the structure and dynamics of lipid membranes. At present, due to the limitations of equipment and conditions, experiments can only indirectly infer the microscopic process of electroporation through macroscopic phenomena and cannot directly observe the occurrence of nanoscale perforation. Therefore, the emergence of molecular dynamics simulation is consequential in the sense that it can compensate for the lack of experiments.

**Fig. 6:**
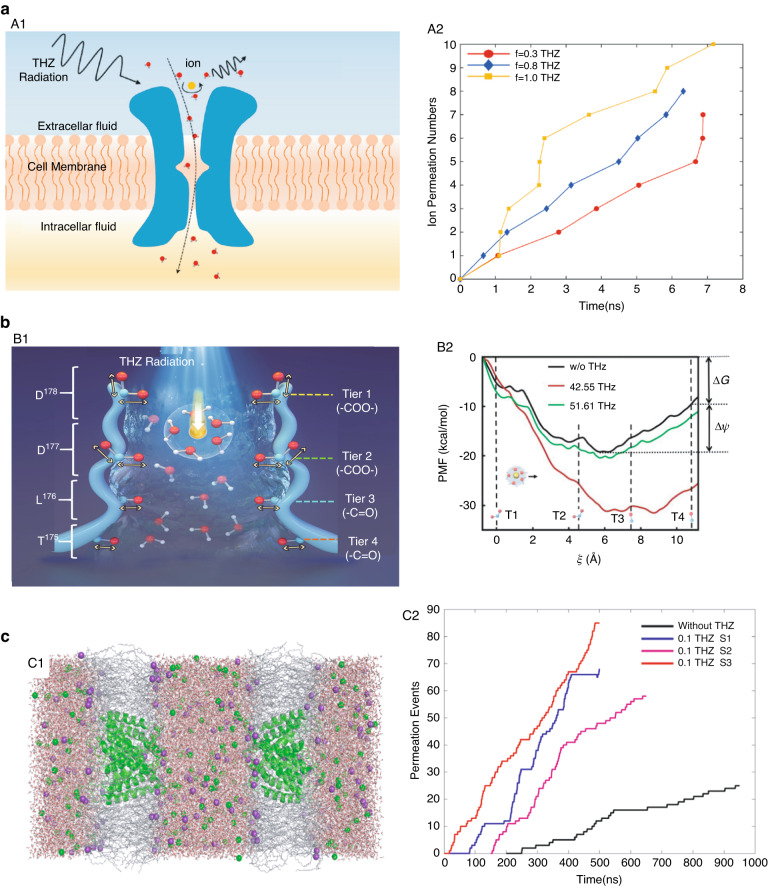
Theoretical calculation and simulation of the bioeffects of terahertz radiation on the nervous system. **a** Theoretical analysis of the effect of terahertz waves on Ca^2+^ transport. A1. Schematic diagram of terahertz waves regulating calcium ion transport across membranes and ions producing terahertz radiation; A2. The variation of ion transmembrane number with time under the control of THz pulses of different frequencies. Reproduced with permission from Ref. ^79^. Copyright 2022, Elsevier. **b** Theoretical analysis of the permeability of the voltage-gated calcium channel when irradiated with terahertz waves. B1. Schematic of accelerated Ca^2+^ permeation through the calcium channel under terahertz radiation; B2. Free energy profiles with and without the presence of external terahertz fields at different frequencies. Reproduced with permission from^80^. Copyright 2021, ACS Publications. **c** Theoretical analysis of the enhancement of K^+^ channel permeability under irradiation with terahertz waves. C1. The simulation system consists of two membranes. C2. Permeation events as a function of time under irradiation. Reproduced with permission from ref. ^81^. Copyright 2022, American Physical Society

Vernier^76^ conducted a study to analyze the terahertz response mechanism of neurons to electrical stimulation. The results showed that the electrical impulses induced changes in neuronal action potentials and transient changes in calcium channels on the cell membrane. Complementary molecular dynamics simulations of the phospholipid bilayer in an electric field confirmed that the response time of water dipoles within the cell membrane to a permeable potential is less than 1 ps, and they are aligned along the electric field direction and redirected at terahertz frequency to achieve electric field inversion. On that basis, Gong^77–79^ established a mathematical physical model of the Ca^2+^ channel by the Brownian dynamics method (Fig. 6a). Under external terahertz field irradiation, the oscillation spectrum of Ca^2+^ is significantly enhanced. Voltage-gated Ca^2+^ channels are activated when the membrane potential exceeds the threshold potential for activation of voltage-gated Ca^2+^ channels during vibration. Fan^80^ focused on the effect of terahertz electromagnetic fields on the permeation of calcium channels. By means of molecular dynamics (MD) simulations, they demonstrated that an external terahertz wave could resonate with the symmetric stretching mode of the −COO− group and cause greatly enhanced permeation of the calcium channel (Fig. 6b). Zhang^81^ studied the effects of terahertz radiation on the K^+^ ion channel (Fig. 6c). Simulation results show that K^+^ ions in the selectivity filter (SF) could respond effectively to certain terahertz waves (0.1 THz) and obtain significant momentum along the direction of the channel, leading to a significant increase in the channel permeation rate. In the case of low power, a long exposure can have the same effect, but if the frequency of radiation changes, it will not produce the effect.

These studies lay the basis for understanding the bioeffects of terahertz irradiation on transmembrane ion transport and pave the way for further exploration of the modulation of intracellular ion concentrations with terahertz electromagnetic waves. At present, theoretical research on the mechanism of terahertz neural regulation is still in its infancy, and there is no unified conclusion. The internal environment of animals is complex, and the structure of neurons is diverse. Theoretical simulation has limitations in accurately reflecting the regulatory mechanism of terahertz waves. In future research, we should further develop simulation models and theoretical algorithms by combining in vitro and in vivo animal experiments.

## Conclusions and perspectives

The application of terahertz technology in the area of neuroscience is a technological innovation that will bring new detection and regulation methods and has great application potential. Currently, the important academic value and immense application prospects of terahertz neuromodulation are gradually being recognized, and its unique advantages are motivating researchers around the world to conduct pioneering interdisciplinary research on biomedical applications of terahertz radiation. Previous studies have accumulated a great deal of data and made some progress in building a theoretical understanding. However, these research results have limitations.

### Key issues and main challenges

First, terahertz neuromodulation is an interdisciplinary subject, and experts from various fields, such as information science, electronics technology, and neuroscience, need to be gathered to detect and analyze the bioeffects. Currently, most of the scholars conducting related research are in neuroscience and life science; this area needs additional attention from experts in the area of information science. In addition, there are only a few teams conducting research on the effects of terahertz neuromodulation, each with its own specific research interest, and the few results that have been published are unsystematic, making it difficult to replicate the results of the research.

Second, the ultimate goal of terahertz neuromodulation is to regulate or stimulate the human brain. There is a great need to carry out in-depth research on the biological safety of terahertz radiation and conduct repeated exposure experiments in live animals to establish appropriate biosafety standards for terahertz radiation. Many existing studies have shown that terahertz radiation does not have safety risks when used for neuromodulation and has good prospects for clinical trials. However, some studies^43^ have also shown that terahertz radiation can cause cytotoxic effects on some cell types. To minimize the harmful effects of terahertz waves, additional detailed studies are needed to clarify the parameters of terahertz waves that can be used for neuromodulation and the types of neurons in which they are effective. Experiments on terahertz radiation effects on large animals, such as primates or humans, should be conducted.

Third, terahertz waves have a wide variety of sources and a wide electromagnetic band. Different radiation parameters produce different biological effects, and some studies have even shown that for the same substance, different terahertz waves produce opposite effects. Therefore, the experimental conditions for research on the biological effects of terahertz waves need to be clarified. On the other hand, different biological tissues respond differently to the same terahertz radiation.

Finally, in addition to electrophysiology, brain neural signals also include chemical signals in the form of neurotransmitters. Conducting research on cellular-level terahertz neuromodulation requires new micro- and nanoscale detection devices. After many years of development, brain–computer interfaces (BCIs) have been used for detection in rodents and primates in vivo, solving the technical problem of detecting bimodal nerve signals at the level of living cells^63,64,66,67,82,83^. Based on current micro/nanoprocessing technology, new microdevices integrated with terahertz fibers and micro/nano brain–computer interfaces can be fabricated. However, to realize the real-time detection of micro-scale neural signals in conscious animals, it is necessary to carry out targeted innovative research in BCI design, processing technology and electronic interfaces to further improve the long-term implant stability, comfort and applicability of brain–computer interface devices.

### Further outlooks and possible breakthroughs

The use of terahertz waves for brain science will open a novel technical pathway for the regulation and detection of the nervous system and assume a commanding presence on the frontiers of science and technology. Experts from various fields, such as information science, electronics technology, and neuroscience, are focusing on this area and have begun to collaborate on related research. With the rapid development of terahertz technology, the price of terahertz radiation sources is decreasing, and an increasing number of teams will soon carry out related research.

Advances in brain–computer interfaces provide new tools for research on terahertz neuromodulation. Brain neural signals include not only electrophysiology but also chemical signals in the form of neurotransmitters. Conducting research on cellular-level terahertz neuromodulation will require new micro- and nanoscale detection devices. After many years of development, brain–computer interfaces (BCIs) have been used for detection in rodents and primates in vivo, solving the technical problem of detecting bimodal nerve signals at the level of living cells^63,64,66,67,82,83^. Based on current micro/nanoprocessing technology, new microdevices integrated with terahertz fibers and micro/nanoscale brain–computer interfaces can be fabricated. This ensures that the terahertz waves are directed to the desired brain tissue, which can solve the problem of poor penetration of terahertz waves into biological tissues.

In vitro neural microelectrode array chips are a new neuronal research tool on which neurons can be cultured as envisaged in some previous works^84,85^. For example, when microgrooves are present, the axons of neurons grow along the microgrooves and then connect with neurons on the other side to form a complete neuron-to-neuron connection; the axons in the microgrooves are structurally complete, and neuronal cytosol is present on both sides of the microgrooves. Through an optical fiber, we can achieve precise stimulation of the desired parts of neurons. Various changes in neurons can be observed after stimulation. In this case, the structure of the neurons is clear and can be detected in individual cells, making it possible to build a neural connection model for theoretical analysis and then verify this model based on the results of actual detection.

In summary, research on the effects of terahertz radiation on the nervous system is a representative of the frontier at the intersection of information and life sciences, with great strategic significance and broad application prospects. Related research can help clarify the mechanisms of neural information encoding and decoding, thus promoting the development of brain science and the treatment of neurological diseases, which has important application prospects in the fields of brain science, information science, life science and national security.
